# Supplementary material for: From Modules to Networks: a Systems-Level Analysis of the Bacitracin Stress Response in Bacillus subtilis
Source: mSystems. 2020 Feb 4;5(1):e00687-19. doi: 10.1128/mSystems.00687-19 (PMC7002115; doi:10.1128/mSystems.00687-19)
Supplement: TABLE S1 [file mSystems.00687-19-st001.docx]

***Supplementary Table S1.***

| **Name** | **Description ^a^** | **Source** |
| --- | --- | --- |
| **TMB1619** | W168 *sacA*::pCHlux103 (P*_bceA_-lux*) | C. Höfler, J. Heckmann, A. Fritsch, P. Popp, S. Gebhard, G. Fritz, and T. Mascher, Microbiology (Reading, Engl) 162:164–176, 2016, <https://doi.org/10.1099/mic.0.000176> |
| **TMB1620** | W168 *sacA*::pCHlux104 (P*_bcrC_-lux*) | C. Höfler, J. Heckmann, A. Fritsch, P. Popp, S. Gebhard, G. Fritz, and T. Mascher, Microbiology (Reading, Engl) 162:164–176, 2016, <https://doi.org/10.1099/mic.0.000176> |
| **TMB1623** | W168 *bceAB*::kan *sacA*::pCHlux103 (P*_bceA_-lux*) | J. Radeck, S. Gebhard, P.S. Orchard, M. Kirchner, S. Bauer, T. Mascher, and G. Fritz, *Mol Microbiol* 100:607–620, 2013 <https://doi.org/10.1111/mmi.13336> |
| **TMB1624** | W168 *bceAB*::kan *sacA*::pCHlux104 (P*_bcrC_-lux*) | J. Radeck, S. Gebhard, P.S. Orchard, M. Kirchner, S. Bauer, T. Mascher, and G. Fritz, *Mol Microbiol* 100:607–620, 2013 <https://doi.org/10.1111/mmi.13336> |
| **TMB1627** | W168 *bcrC*::tet *sacA*::pCHlux103 (P*_bceA_-lux*) | J. Radeck, S. Gebhard, P.S. Orchard, M. Kirchner, S. Bauer, T. Mascher, and G. Fritz, *Mol Microbiol* 100:607–620, 2013 <https://doi.org/10.1111/mmi.13336> |
| **TMB1628** | W168 *bcrC*::tet *sacA*::pCHlux104 (P*_bcrC_-lux*) | J. Radeck, S. Gebhard, P.S. Orchard, M. Kirchner, S. Bauer, T. Mascher, and G. Fritz, *Mol Microbiol* 100:607–620, 2013 <https://doi.org/10.1111/mmi.13336> |
| **TMB1632** | W168 *bceAB*::kan *bcrC*::tet *sacA*::pCHlux104 (P*_bcrC_-lux*) | This study |
