# Supplementary material for: From Modules to Networks: a Systems-Level Analysis of the Bacitracin Stress Response in Bacillus subtilis
Source: mSystems. 2020 Feb 4;5(1):e00687-19. doi: 10.1128/mSystems.00687-19 (PMC7002115; doi:10.1128/mSystems.00687-19)
Supplement: TEXT S1 [file mSystems.00687-19-s0001.docx]

**Supplementary Text to**

**From modules to networks: A systems-level analysis of the bacitracin stress response in *B. subtilis***

Hannah Piepenbreier^1^, Andre Sim^1^, Carolin M. Kobras^2^, Jara Radeck^3^, Thorsten Mascher^3^, Susanne Gebhard^2^ and Georg Fritz^1,4,^*

*^1^LOEWE Center for Synthetic Microbiology and Department of Physics,* *Philipps-Universität Marburg, Germany*; *^2^Department of Biology & Biochemistry, Milner Centre for Evolution, University of Bath, United Kingdom; ^3^Institute of Microbiology, Technische Universität (TU) Dresden, 01062 Dresden, Germany*; *^4^Present address: School of Molecular Sciences, The University of Western Australia, Perth, Western Australia 6009, Australia*

*For correspondence: Email: [georg.fritz@uwa.edu.au](mailto:georg.fritz@uwa.edu.au); Tel: +61 8 6488 3142

***Computational model of the bacitracin stress response network***

To build the computational model of the bacitracin stress response network, we combined the pre-existing mathematical description of the lipid II cycle (1) and the previously developed theory of the BceAB transporter production in response to bacitracin (2) and included a description of the BcrC module in addition.

The kinetic model of the lipid II cycle (1) describes the time-dependent changes of the concentrations of the different lipid II cycle intermediates (as illustrated in Fig. 1 in the main text) by deterministic differential equations to monitor the dynamics of cell wall biosynthesis per individual cell. The well-studied enzymatic reactions of MraY, MurG, the diverse penicillin-binding protein (PBPs) and the two undecaprenyl pyrophosphate phosphatases (UppPs) were modelled by Michaelis-Menten kinetics, for which substrate levels (*S_i_*), enzyme levels (*E*), catalytic constants of the enzymes (*k_cat_*) as well as the Michaelis-Menten constants (*K_M_*) parameterize the reaction dynamics. Since the biochemical properties of the enzymes catalysing the flipping reaction of lipid II were largely unknown and the flipping of UPP and UP were only hypothesized, the model describes these reactions by first order kinetics, as quantified by an effective rate constant *k_i_* (*i = UP, UPP, LII*). Furthermore, growth-driven dilution of all lipid II cycle intermediates occurring at a constant rate *γ* was integrated in the model of the lipid II cycle. This dilution was assumed to be counterbalanced by the constant *de novo* synthesis of UPP in the cytoplasm by rate *α*. Additionally, the previous model comprised a theoretical description of cell wall antibiotic treatment. In particular, the interaction between an antibiotic and its target within the lipid II cycle was described as a ligand-binding reaction determined by the *in vitro* dissociation constant (*K_D_*), which is defined as the ratio between the dissociation (*k_diss_*) and association rate (*k_ass_*), respectively. Thus, the following time-dependent model variables result from this described scope of the pre-existing model of the lipid II cycle:

- UPP_IN_ = internal pool of undecaprenyl pyrophosphate (UPP)
- UPP_OUT_ = external pool of undecaprenyl pyrophosphate
- UP_IN_ = internal pool of undecaprenyl phosphate (UP)
- UP_OUT_ = external pool of undecaprenyl phosphate
- LI = pool of lipid I
- LII_IN_ = internal pool of lipid II
- LII_OUT_ = external pool of lipid II
- UPP-BAC = pool of bacitracin-bound UPP

The existing theory of the BceAB resistance module (2) describes the regulatory dynamics of the Bce system in response to bacitracin in detail. Here, the BceAB transporter was assumed to catalyse the release of bacitracin from UPP with Michaelis-Menten enzyme kinetics. Furthermore, the model comprised a detailed description of the production of new BceAB transporter in response to bacitracin, which is governed by a special flux-sensing mechanism. In particular, it was proposed that the production of new BceAB transporter is adapted to the capacity of the cell to deal with the present amount of bacitracin – monitored via the load of UPP-BAC per existing BceAB transporter. Accordingly, the load of UPP-BAC per transporter - called *J_BAC_* – was assumed to dictate the BceAB synthesis. While the sensing and signalling process via the TCS BceRS was not modelled in detail, the activation of transporter production was assumed to be directly proportional to *J_BAC_*. To describe the synthesis process of BceAB based on the flux sensing mechanism in detail, both the processes of transcription and translation were taken into account. Hence, the transcriptional synthesis of *bceAB* mRNA was described as a function of *J_BAC_* by following a thermodynamic model for translational regulation. Here, the concentration of *bceAB* mRNA is dependent on a basal transcription rate (*α*), the ratio of maximal to basal promoter activity (*ω*), the relative load per transporter at which P*_bceA_* is activated (*κ*) and the mRNA degradation rate (*λ*). Finally, the concentration of the BceAB transporter depends on the translation rate per mRNA (*β*) and the protein dilution due to cell growth. However, the model aimed to quantitatively describe the results of measurements of the P*_bceA_-luxABCDE* reporter, which illustrates the activation of BceAB production in response to bacitracin. Thus, the dynamic equations quantifying the Lux protein production were formulated analogously to the described assumptions above and a multiplicative scaling factor was introduced to relate the Lux protein levels to the experimentally measured luminescence output (*δ*). Hence, from this comprehensive description of the BceAB resistance module additional model variables arise:

- m_BceAB_ = *bceAB* mRNA
- BceAB = pool of BceAB transporter
- m_LUX_ = *luxABCDE* mRNA
- Lux = pool of Lux proteins
- lumi = levels of luminescence (Lux activity)

Finally, to describe the BcrC resistance module, we introduced a scaling factor (*s^BcrC^*) for the reaction rate of UPP dephosphorylation, which reflects the increase in BcrC levels in response to bacitracin (as described in detail in the main text). Considering the contribution of BcrC and UppP to the overall phosphatase activity, the scaling factor was calculated as follows:

$$s^{BcrC}=x^{BcrC}*f^{BcrC}(BAC)+[1-x^{BcrC}]*1$$

Here, *x^BcrC^ ϵ [0;1]* describes the contribution of BcrC to the overall phosphatase activity and (*1- x^BcrC^*) the contribution of UppP, respectively. Furthermore, $f^{BcrC}(BAC)$ displays the bacitracin-dependent fold-change in BcrC levels between no bacitracin treatment and a certain bacitracin concentration (derived from the P*_bcrC_* activity, as explained in the main text). In addition, we aimed to integrate the observation that the total concentration of lipid intermediates increases when BcrC is lacking. To this end, we introduced another scaling factor (*s^UPP^*) for the *de novo* synthesis of lipid intermediates in the form of UPP, which was set to 1 in the wild-type scenario.

In the end, when taking all the individual parts of the bacitracin stress response network into account, the model equations that quantify the time-dependent changes of the concentrations of lipid II cycle intermediates and BceAB were formulated as follows:

| $\frac{d\left[ UPP_{IN} \right]}{dt}= s^{UPP}* \alpha^{UPP}-k_{UPP}\left[ UPP_{IN} \right]- \gamma[UPP_{IN}]$ | (I) |
| --- | --- |
| $\frac{d\left[ UPP_{OUT} \right]}{dt}= k_{UPP}\left[ UPP_{IN} \right]-s^{BcrC}* v_{max}^{UppPs}\frac{\left[ UPP_{OUT} \right]}{K_{M}^{UppPs}+\left[ UPP_{OUT} \right]}+v_{max}^{PBPs}\frac{\left[ {LII}_{OUT} \right]}{K_{M}^{PBPs}+\left[ {LII}_{OUT} \right]}-k_{ass}^{BAC;UPP}\left[ UPP_{OUT} \right]\left[ BAC \right]+k_{diss}^{BAC;UPP}\left[ UPP-BAC \right]+k_{cat}^{BceAB} \left[ BceAB \right] J_{BAC}- \gamma[UPP_{OUT}]$ | (II) |
| $\frac{d\left[ UP_{OUT} \right]}{dt}= {s^{BcrC}* v}_{max}^{UppPs}\frac{\left[ UPP_{OUT} \right]}{K_{M}^{UppPs}+\left[ UPP_{OUT} \right]}-k_{UP}[UP_{OUT}]- \gamma[UP_{OUT}]$ | (III) |
| $\frac{d\left[ UP_{IN} \right]}{dt}= k_{UP}[UP_{OUT}]{- v}_{max}^{MraY}\frac{\left[ UP_{IN} \right]}{K_{M}^{MraY}+\left[ UP_{IN} \right]}- \gamma[UP_{IN}]$ | (IV) |
| $\frac{d\left[ LI \right]}{dt}=v_{max}^{MraY}\frac{\left[ UP_{IN} \right]}{K_{M}^{MraY}+\left[ UP_{IN} \right]}-v_{max}^{MurG}\frac{\left[ LI \right]}{K_{M}^{MurG}+\left[ LI \right]}- \gamma[LI]$ | (V) |
| $\frac{d\left[ LII_{IN} \right]}{dt}=v_{max}^{MurG}\frac{\left[ LI \right]}{K_{M}^{MurG}+\left[ LI \right]}-k_{LII}[LII_{IN}]- \gamma[LII_{IN}]$ | (VI) |
| $\frac{d\left[ LII_{OUT} \right]}{dt}=k_{LII}\left[ LII_{IN} \right]-v_{max}^{PBPs}\frac{\left[ {LII}_{OUT} \right]}{K_{M}^{PBPs}+\left[ {LII}_{OUT} \right]}- \gamma[LII_{OUT}]$ | (VII) |
| $\frac{d\left[ UPP-BAC \right]}{dt}=k_{ass}^{BAC;UPP}\left[ UPP_{OUT} \right]\left[ BAC \right]-k_{diss}^{BAC;UPP}\left[ UPP-BAC \right]-k_{cat}^{BceAB} \left[ BceAB \right] J_{BAC} - \gamma[UPP-BAC]$ | (VIII) |
| $\frac{d\left[ m_{BceAB} \right]}{dt}=\alpha^{BceAB} \left( \frac{1+\omega\left( \frac{J_{BAC}}{\kappa} \right)^{n}}{1+\left( \frac{J_{BAC}}{\kappa} \right)^{n}} \right)-\lambda^{BceAB} \left[ m_{BceAB} \right]$ | (IX) |
| $\frac{d\left[ BceAB \right]}{dt}=\beta\left[ m_{BceAB} \right]-\gamma\left[ BceAB \right]$ | (X) |
| $\frac{d\left[ m_{Lux} \right]}{dt}=\alpha^{BceAB} \left( \frac{1+\omega\left( \frac{J_{BAC}}{\kappa} \right)^{n}}{1+\left( \frac{J_{BAC}}{\kappa} \right)^{n}} \right)-\lambda^{Lux} \left[ m_{Lux} \right]$ | (XI) |
| $\frac{d\left[ Lux \right]}{dt}=\beta\left[ m_{Lux} \right]-\gamma^{Lux}\left[ Lux \right]$ | (XII) |
| $lumi =\delta\left[ Lux \right]$ | (XIII) |

with $J_{BAC}=\frac{\frac{\left[ UPP-BAC \right]}{K_{M}^{BceAB}}}{1 + \frac{\left[ UPP-BAC \right]}{K_{M}^{BceAB}}}$.

To study the effect of bacitracin on the progression of the lipid II cycle, we monitored the effect of bacitracin on the rate of PG synthesis, *j_PG_*, which was formulated as follows

| $j_{PG}= v_{max}^{PBPs}\frac{\left[ {LII}_{OUT} \right]}{K_{M}^{PBPs}+\left[ {LII}_{OUT} \right]}$ | (XIV) |
| --- | --- |

***Model adaptations to describe the mutant strains***

The full model described above reproduces the scenario of a wild-type strain where both resistance determinants (BceAB and BcrC) are fully intact. However, adaptations were necessary to simulate a lack of one or both of the two resistance modules. In order to describe the scenario of a *ΔbceAB* mutant, we set the basal production rate of BceAB to zero (*α^BceAB^* = 0) to avoid BceAB and Lux production. Furthermore, we adapted the fold-change *f^BcrC^* to the reporter output of P*_bcrC_-luxABCDE* in a *ΔbceAB* mutant. Secondly, we simulated a lack of BcrC, as in a *ΔbcrC* mutant, by setting the contribution of BcrC to the overall phosphatase activity to zero (*x^BcrC^* = 0). In addition, the scaling factor for the production rate of UPP was adapted (*s^UPP^* >1), since lipid carrier production was assumed to be up-regulated in response to *bcrC* deletion (see qPCR data in the main text). The deviation of the precise value for this parameter is explained in the section below. Finally, to study a lack of both resistance modules (*ΔbceABΔbcrC* mutant), we combined the two adaptations for the single mutants (*α^BceAB^* = 0, *x^BcrC^* = 0 and *s^UPP^* >1).

***Calibration of the mathematical model***

In order to calibrate the model, we aimed to identify physiologically relevant values for the parameters in Eqs. (I-XIV). At first, we set all known parameters from the model of the lipid II cycle to its previously defined values (see Supplementary Table 3).

Subsequently, we determined the new parameters arising from the mathematical description of the BcrC resistance module, namely *x^BcrC^*, which defined *s^BcrC^*, and *s^UPP^*, respectively, within the model. For this purpose, we compared the scenarios of a strain lacking both resistance modules (*ΔbceABΔbcrC* mutant) or featuring the BcrC resistance module solely (*ΔbceAB* mutant) from a theoretical point of view: Each of the two parameters affects both the progression of the lipid II cycle without bacitracin and the effect of bacitracin treatment on the lipid II cycle. While a higher impact of BcrC on the overall phosphatase activity supports the progression of the cycle more efficiently when BcrC is present, the rate of PG synthesis would be reduced more strongly in this case when BcrC is lacking. This demands a more pronounced upregulation of the production of lipid II cycle intermediates in response to *bcrC* deletion to recover a close-to-optimal PG synthesis rate. However, the model also predicted a PG synthesis rate above the optimal one when assuming an excessive upregulation of lipid carrier production, which is not valid in a physiological sense. Furthermore, variations in the PG synthesis rate without bacitracin treatment clearly imply significant differences in the amount of bacitracin cells can stand. Obviously, in the scenario where BcrC is present as a resistance module (*ΔbceAB* mutant), a stronger contribution of BcrC to the overall phosphatase activity confers higher resistance and coincides with a raised IC_50_. However, when lacking BcrC (*ΔbceABΔbcrC* mutant), the PG synthesis rate without bacitracin treatment dictates the susceptibility towards bacitracin. If the PG synthesis rate is still distinctly affected in the untreated scenario, little amounts of bacitracin would be sufficient to reduce the PG synthesis rate to half of its optimum. In contrast, much higher bacitracin concentrations are required to reach 50% of the optimal PG synthesis rate when the rate is nearly unaffected without antibiotic. Thus, as the PG synthesis rate without bacitracin treatment is governed by the contribution of BcrC on the overall phosphatase activity (*x^BcrC^*) and lipid carrier upregulation in response to BcrC shortage (*s^UPP^*) – as explained above – the IC_50_ prediction of the model for the *ΔbceABΔbcrC* mutant strongly depends on these two parameters. We ultimately aimed to find a theoretical model that simultaneously describe the progression of the lipid II cycle with (*ΔbceAB*) and without BcrC (*ΔbcrCΔbceAB*) precisely and matches physiological conditions as well. Therefore, we simulated the IC_50_ model predictions for 50x50 combinations of the two parameters *x^BcrC^* and *s^UPP^* and determined the weighted squared 2-norm *χ^2^* for all possible combinations as follows:

$$\chi^{2}(x^{BcrC},s^{UPP})=\frac{\left( IC_{50}^{\Delta bceAB}\left( x^{BcrC},s^{UPP} \right)-MIC^{\Delta bceAB} \right)^{2}}{\left( \sigma_{MIC^{\Delta bceAB}} \right)^{2}} +\frac{\left( IC_{50}^{\Delta bceAB\Delta bcrC}\left( x^{BcrC},s^{UPP} \right)-MIC^{\Delta bceAB\Delta bcrC} \right)^{2}}{\left( \sigma_{MIC^{\Delta bceAB\Delta bcrC}} \right)^{2}}$$

Here, $MIC^{\Delta bceAB}$ and $MIC^{\Delta bceAB\Delta bcrC}$ represent the experimentally determined MICs in the different strains and $\sigma_{MIC^{\Delta bceAB}}$and $\sigma_{MIC^{\Delta bceAB\Delta bcrC}}$ the respective errors in the experimental MICs, calculated by the error propagation formula (errors are given in the main text). Furthermore, *IC_50_^ΔbceAB^* and *IC_50_^ΔbceABΔbcrC^* describe the model-predicted IC_50_^’^s, dependent on the parameters *x^BcrC^* and *s^UPP^*.

To find the optimal parameter combination, we demanded the following two constraints for the two model parameters:

1. $\chi^{2}\left( x^{BcrC},s^{UPP} \right)\to min$

and

1. $j_{PG}^{\Delta bceAB\Delta bcrC}(x^{BcrC},s^{UPP})\leq j_{PG}^{WT}$

*j_PG_^WT^* and *j_PG_^ΔbceABΔbcrC^* display the theoretical rates of PG synthesis without bacitracin treatment in the wild-type scenario and a scenario where both resistance modules are lacking, respectively. The second constraint accounts for the physiological plausible limitation of the PG synthesis rate to its wild-type level. In Supplementary Figure S2, the *χ^2^* values are plotted against the parameter combinations. Standard deviations on the two model parameters were determined from the 68.3 % confidence intervals as described (3) and also illustrated in Supplementary Figure S2. The final parameter values of *x^BcrC^* and *s^UPP^* as well as their standard deviation σ are given in Supplementary Table 3).

Finally, we determined the parameters originating from the previous model of the BceAB resistance module. Since the setup of the experimental measurements of the expression levels of the resistance modules, which we now aimed to quantitatively describe by the new model, was quite different from the previous experimental study that was used to calibrate the pre-existing model, we were not able to transfer the existing parameters to our new model. Rather, significant variations in the growth conditions between both experimental approaches demanded adaptations in the model parameters that describe the dynamics of the Bce system theoretically. Therefore, we fixed the model parameters that are independent from the growth conditions (e.g. mRNA degradation rates, translation rate) to their pre-defined, physiological values and determined the remaining ones by a constrained optimization approach. Here, the experimental data of the P*_bceAB_-luxABCDE* reporter output provide nine objectives to the seven unknown model parameters. To solve this over-determined non-linear data-fitting problem, we used the solving function *lsqnonlin*, embedded in the MATLAB^TM^ software. This function solves nonlinear least-square curve fitting problems of the form

$$min\left\| f\left( x \right) \right\|_{2}^{2}=min(f_{1}\left( x \right)^{2}+f_{2}\left( x \right)^{2}+\ldots+f_{n}\left( x \right)^{2})$$

by using a trust-region reflective Newton algorithm. As outputs, it returns the optimal parameter set $\bar{x}$ of the problem as well as the squared-2 norm *χ^2^* of the residual at $\bar{x}$ ($\chi^{2}= \sum f\left( \bar{x} \right)^{2}$). To account for the presence of local optima, 50 independent fits were performed with randomly chosen initial parameter sets and the best-fit result was given at minimal *χ^2^*. The optimal parameters are shown in Supplementary Table S3. We followed (4) to compute the asymmetric errors σ_+_ and σ_-_ with respect to the optimal parameter values $\bar{x}$, listed in Supplementary Table S3. The squared errors for the parameter $x_{k}$ were calculated using the following equations:

$$\sigma_{k,+}^{2}= \frac{\sum_{i:x_{k,i}>\bar{x}_{k}} \left( x_{k,i}- \bar{x_{k}} \right)^{2}e^{-\chi_{i}^{2}/2}}{\sum_{i:x_{k,i}>\bar{x}_{k}} e^{-\chi_{i}^{2}/2}}$$

and

$$\sigma_{k,-}^{2}= \frac{\sum_{i:x_{k,i}<\bar{x}_{k}} \left( x_{k,i}- \bar{x_{k}} \right)^{2}e^{-\chi_{i}^{2}/2}}{\sum_{i:x_{k,i}<\bar{x}_{k}} e^{-\chi_{i}^{2}/2}}$$

Where $x_{k,i}$ is the value of the parameter $x_{k}$in the i^th^ fit, $\bar{x}_{k}$ is the value of $x_{k}$ in the fit with the lowest value of $\chi^{2}$, and $\chi_{i}^{2}$ is the value of $\chi^{2}$ for the i^th^ fit. In using the likelihood function $e^{-\chi_{i}^{2}/2}$, we assumed that the errors in the measurements are independent and normally distributed with widths equal to the standard error of the mean.

***Model modification***

In order to study the futile activation of BceAB production by UPP (as explained in detail in the main text), we slightly modified the model description of the BceAB resistance module. According to the assumption that UPP affects the transporter state by futile binding, we expected the load per transporter dependent from both UPP and UPP-BAC, which were assumed to bind in a competitive manner. Thus, we adapted the description of the load per transporter, which affects the activation of BceAB production, and introduced a modified description of transporter load *J_load_*:

$$J_{load}=J_{BAC}^{'}+J_{futile}$$

with

$$J_{BAC}^{'} = \frac{\frac{\left[ UPP-BAC \right]}{K_{M}^{BceAB}}}{1 + \frac{\left[ UPP-BAC \right]}{K_{M}^{BceAB}} + \frac{\left[ UPP \right]}{\tilde{K}_{M}^{BceAB}}}$$

and

$$J_{futile} =\frac{\frac{\left[ UPP \right]}{\tilde{K}_{M}^{BceAB}}}{1 + \frac{\left[ UPP-BAC \right]}{K_{M}^{BceAB}} + \frac{\left[ UPP \right]}{\tilde{K}_{M}^{BceAB}}},$$

leading to

$$J_{load} =\frac{\frac{\left[ UPP-BAC \right]}{K_{M}^{BceAB}}+ \frac{\left[ UPP \right]}{\tilde{K}_{M}^{BceAB}}}{1 + \frac{\left[ UPP-BAC \right]}{K_{M}^{BceAB}} + \frac{\left[ UPP \right]}{\tilde{K}_{M}^{BceAB}}},$$

Here, the Michaelis constants $\tilde{K}_{M}^{BceAB}$and $K_{M}^{BceAB}$ describe the binding affinities of UPP and UPP-Bac to the transporter, respectively. Since the load per transporter affects the activation of BceAB and Lux protein production, the respective two model Eqs. (IX) and (XI) were changed as follows:

| $\frac{d\left[ m_{BceAB} \right]}{dt}=\alpha^{BceAB} \left( \frac{1+\omega\left( \frac{J_{load}}{\kappa} \right)^{n}}{1+\left( \frac{J_{load}}{\kappa} \right)^{n}} \right)-\lambda^{BceAB} \left[ m_{BceAB} \right]$ | (XV) |
| --- | --- |
| $\frac{d\left[ m_{Lux} \right]}{dt}=\alpha^{BceAB} \left( \frac{1+\omega\left( \frac{J_{load}}{\kappa} \right)^{n}}{1+\left( \frac{J_{load}}{\kappa} \right)^{n}} \right)-\lambda^{Lux} \left[ m_{Lux} \right]$ | (XVI) |

However, we expected that futile binding of UPP inactivates the transporter but does not provoke any reaction. Therefore, the rate of release of bacitracin from UPP is solely dependent on $J_{BAC}^{'}$:

| $\frac{d\left[ UPP_{OUT} \right]}{dt}= k_{UPP}\left[ UPP_{IN} \right]-s^{BcrC}* v_{max}^{UppPs}\frac{\left[ UPP_{OUT} \right]}{K_{M}^{UppPs}+\left[ UPP_{OUT} \right]}+v_{max}^{PBPs}\frac{\left[ {LII}_{OUT} \right]}{K_{M}^{PBPs}+\left[ {LII}_{OUT} \right]}-k_{ass}^{BAC;UPP}\left[ UPP_{OUT} \right]\left[ BAC \right]+k_{diss}^{BAC;UPP}\left[ UPP-BAC \right]+k_{cat}^{BceAB} \left[ BceAB \right] {J'}_{BAC}- \gamma[UPP_{OUT}]$ | (XVII) |
| --- | --- |
| $\frac{d\left[ UPP-BAC \right]}{dt}=k_{ass}^{BAC;UPP}\left[ UPP_{OUT} \right]\left[ BAC \right]-k_{diss}^{BAC;UPP}\left[ UPP-BAC \right]-k_{cat}^{BceAB} \left[ BceAB \right] {J'}_{BAC} - \gamma[UPP-BAC]$ | (XVIII) |

After model modification, we proved that the predictions the IC_50_s remain unaffected from the modifications.

**Supplementary References**

1. Piepenbreier H., Diehl A., and Fritz G. (2019), Minimal exposure of lipid II cycle intermediates triggers cell wall antibiotic resistance. *Nature Communications* **10**: 2733
2. Fritz, G., Dintner, S., Treichel, N.S., Radeck, J., Gerland, U., Mascher, T., and Gebhard, S. (2015) A new way of sensing: Need-based activation of antibiotic resistance by a flux-sensing mechanism. *mBio* **6**: e00975.
3. Press, W.H., Teukolsky, S.A., Vetterling, W.T., and Flannerty, B.P. (1992) *Numerical Recipes in C: The Art of Scientific Computing.* New York: Cambridge University Press.
4. Wall, M.E., Markowitz, D.A., Rosner, J.L., and Martin, R.G. (2009) Model of transcriptional activation by MarA in *Escherichia coli*. arXiv:0902.0959v1.
