# Supplementary figures and images for: From Modules to Networks: a Systems-Level Analysis of the Bacitracin Stress Response in Bacillus subtilis
Source: mSystems. 2020 Feb 4;5(1):e00687-19. doi: 10.1128/mSystems.00687-19 (PMC7002115; doi:10.1128/mSystems.00687-19)

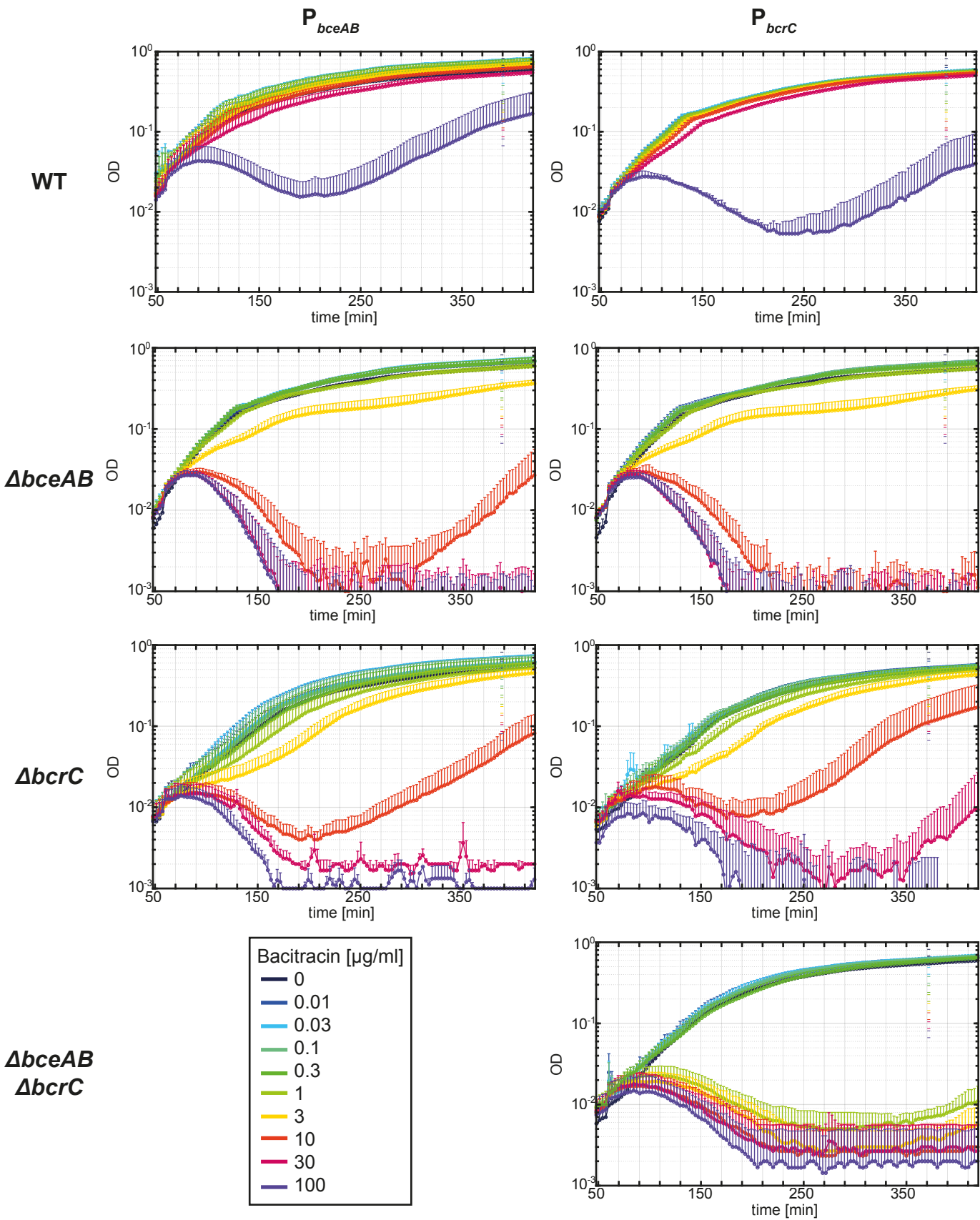

Supplement: FIG S1 [file mSystems.00687-19-sf001.pdf]

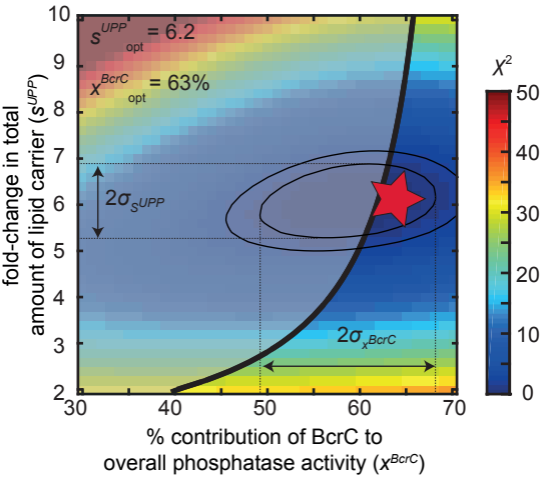

Supplement: FIG S2 [file mSystems.00687-19-sf002.pdf]
