# Supplementary material for: From Modules to Networks: a Systems-Level Analysis of the Bacitracin Stress Response in Bacillus subtilis
Source: mSystems. 2020 Feb 4;5(1):e00687-19. doi: 10.1128/mSystems.00687-19 (PMC7002115; doi:10.1128/mSystems.00687-19)
Supplement: TABLE S2 [file mSystems.00687-19-st002.docx]

***Supplementary Table S2.***

| **Name** | **Description** | **Resistance**  **in *E. coli* /**  ***B. subtilis* ^a^** | **Source** |
| --- | --- | --- | --- |
| **pCHlux103** | pAH328-derivative, *sacA*::P*_bceA_-lux*, *cat*, *bla* | Amp^r^ / cm^r^ | C.Höfler, J. Heckmann, A. Fritsch, P. Popp, S. Gebhard, G. Fritz, and T. Mascher, Microbiology (Reading, Engl) 162:164–176, 2016, <https://doi.org/10.1099/mic.0.000176> |
| **pCHlux104** | pAH328-derivative, *sacA*::P*_bcrC_-lux*, *cat*, *bla* | Amp^r^ / cm^r^ | C.Höfler, J. Heckmann, A. Fritsch, P. Popp, S. Gebhard, G. Fritz, and T. Mascher, Microbiology (Reading, Engl) 162:164–176, 2016, <https://doi.org/10.1099/mic.0.000176> |
