# Supplementary material for: From Modules to Networks: a Systems-Level Analysis of the Bacitracin Stress Response in Bacillus subtilis
Source: mSystems. 2020 Feb 4;5(1):e00687-19. doi: 10.1128/mSystems.00687-19 (PMC7002115; doi:10.1128/mSystems.00687-19)
Supplement: TABLE S3 [file mSystems.00687-19-st003.docx]

**Supplementary Table S3.**

| **Parameter** | **Notation** | **Value** | **Source** | |
| --- | --- | --- | --- | --- |
| LII module | | | | |
| Michaelis-Menten constant for Lipid I synthesis via MraY | $K_{M}^{MraY}$ | 177 $\mu M$ | | H. Piepenbreier, A. Diehl, and G. Fritz, *Nat Commun* 10:2733, 2019, <https://doi.org/10.1038/s41467-019-10673-4> |
| Michaelis-Menten constant for Lipid II synthesis via MurG | $K_{M}^{MurG}$ | 25 $\mu M$ | | H. Piepenbreier, A. Diehl, and G. Fritz, *Nat Commun* 10:2733, 2019, <https://doi.org/10.1038/s41467-019-10673-4> |
| Michaelis-Menten constant for PG synthesis via PBPs | $K_{M}^{PBPs}$ | 18 $\mu M$ | | H. Piepenbreier, A. Diehl, and G. Fritz, *Nat Commun* 10:2733, 2019, <https://doi.org/10.1038/s41467-019-10673-4> |
| Michaelis-Menten constant for UPP dephosphorylation via UppPs | $K_{M}^{UppPs}$ | 4.69 x 10^3^ $\mu M$ | | H. Piepenbreier, A. Diehl, and G. Fritz, *Nat Commun* 10:2733, 2019, <https://doi.org/10.1038/s41467-019-10673-4> |
| Maximal Lipid I synthesis rate via MraY | $v_{max}^{MraY}$ | 3.01 x 10^5^ $\frac{\mathbf{molecules}}{\mathbf{min}}$ | | H. Piepenbreier, A. Diehl, and G. Fritz, *Nat Commun* 10:2733, 2019, <https://doi.org/10.1038/s41467-019-10673-4> |
| Maximal Lipid II synthesis rate via MurG | $v_{max}^{MurG}$ | 3.21 x 10^5^ $\frac{\mathbf{molecules}}{\mathbf{min}}$ | | H. Piepenbreier, A. Diehl, and G. Fritz, *Nat Commun* 10:2733, 2019, <https://doi.org/10.1038/s41467-019-10673-4> |
| Maximal PG synthesis rate via PBPs | $v_{max}^{PBPs}$ | 2.32 x 10^5^ $\frac{\mathbf{molecules}}{\mathbf{min}}$ | | H. Piepenbreier, A. Diehl, and G. Fritz, *Nat Commun* 10:2733, 2019, <https://doi.org/10.1038/s41467-019-10673-4> |
| Maximal UPP dephosphorylation rate via UppPs | $v_{max}^{UppPs}$ | 3.55 x 10^5^ $\frac{\mathbf{molecules}}{\mathbf{min}}$ | | H. Piepenbreier, A. Diehl, and G. Fritz, *Nat Commun* 10:2733, 2019, <https://doi.org/10.1038/s41467-019-10673-4> |
| Reaction rate of UPP flipping | $k_{UPP}$ | 1.84 x 10^3^ $\frac{\mathbf{1}}{\mathbf{min}}$ | | H. Piepenbreier, A. Diehl, and G. Fritz, *Nat Commun* 10:2733, 2019, <https://doi.org/10.1038/s41467-019-10673-4> |
| Reaction rate of UP flipping | $k_{UP}$ | 3.81 $\frac{\mathbf{1}}{\mathbf{min}}$ | | H. Piepenbreier, A. Diehl, and G. Fritz, *Nat Commun* 10:2733, 2019, <https://doi.org/10.1038/s41467-019-10673-4> |
| Reaction rate of LII flipping | $k_{LII}$ | 642.23 $\frac{\mathbf{1}}{\mathbf{min}}$ | | H. Piepenbreier, A. Diehl, and G. Fritz, *Nat Commun* 10:2733, 2019, <https://doi.org/10.1038/s41467-019-10673-4> |
| Production rate of UPP | $\alpha^{UPP}$ | 4.76 x 10^4^ $\frac{\mathbf{molecules}}{\mathbf{min}}$ | | H. Piepenbreier, A. Diehl, and G. Fritz, *Nat Commun* 10:2733, 2019, <https://doi.org/10.1038/s41467-019-10673-4> |
| Dilution rate of all proteins | $\gamma$ | 0.017 $\frac{\mathbf{1}}{\mathbf{min}}$ | | Adapted to cell’s doubling time, $\gamma=\frac{ln2}{T_{D}}$ |
| Antibiotic action | | | | |
| Binding constant for bacitracin – UPP interaction | $K_{D}^{BAC;UPP}$ | 1 µM | | D.R. Storm, J.L. and Strominger, *J Biol Chem* 248:3940–3945, 1973, <http://www.jbc.org/content/248/11/3940> |
| Dissociation rate for bacitracin – UPP interaction | $k_{diss}^{BAC;UPP}$ | 0.75 $\frac{\mathbf{1}}{\mathbf{min}}$ | | Estimated from Fig. 2B in (N.J. Economou, S. Cocklin, and P.J. Loll, *Proc Natl Acad Sci USA* 110:14207–14212, 2013, <https://doi.org/10.1073/pnas.1308268110>) |
| Association rate for bacitracin – UPP interaction | $k_{ass}^{BAC;UPP}$ | 0.75 $\frac{\mathbf{1}}{\mu M \mathbf{min}}$ | | Adjusted to match $K_{D}^{BAC;UPP}$ |
| BceAB module | | | | |
| Michaelis-Menten constant for bacitracin transport via BceAB | $K_{M}^{BceAB}$ | 129 (+137/-59)^a^ $\mu M$ | | Estimated parameter |
| Catalytic efficiency of every BceAB transporter | $k_{cat}^{BceAB}$ | 1.51 (+1.4/-0.2)^a^ x 10^3^ $\frac{\mathbf{molecules}}{\mathbf{transpoter * min}}$ | | Estimated parameter |
| Basal transcription rate of P*_bceAB_* promoter | $\alpha^{BceAB}$ | 2.62 (+1.0/-0.8)^a^ x 10^-2^ $\frac{\mathbf{mRNA}}{\mathbf{min}}$ | | Estimated parameter |
| Fold-change of P*_bceAB_* promoter | $\omega$ | 2.776 (+0.7/-0.7)^a^ x 10^3^ | | Estimated parameter (Suggested by data in Fig. 2B(iii) in the main text) |
| P*_bceAB_* activation threshold | $\kappa$ | 1.3 (+3.0/-0.2)^a^ | | Estimated parameter |
| Hill coefficient (reflects all forms of cooperativity in stimulus perception and signal transduction) | $n$ | 4.7 (+4.0/-1.1)^a^ | | Estimated parameter |
| *bceAB* mRNA degradation rate | $\lambda^{BceAB}$ | 0.462 $\frac{\mathbf{1}}{\mathbf{min}}$ | | Corresponds to a *bceAB* mRNA half-life of 1.5 min (G. Fritz, S. Dintner, N.S. Treichel, J. Radeck, U. Gerland, T. Mascher, and S. Gebhard, *mBio* 6:e00975, 2015, <https://doi.org/10.1128/mBio.00975-15>) |
| *luxABCDE* mRNA degradation rate | $\lambda^{Lux}$ | 0.138 $\frac{\mathbf{1}}{\mathbf{min}}$ | | Corresponds to a *lux* mRNA half-life of 5 min; upper limit for mRNA half-life inferred in (J. Radeck, K. Kraft, J. Bartels, T. Cikovic, F. Duerr, J. Emenegger, S. Kelterborn, C. Sauer, G. Fritz, S. Gebhard, and T. Mascher, J Biol Eng 7:29, 2013, <https://doi.org/10.1186/1754-1611-7-29>) |
| LuxABCDE protein decay rate | $\gamma^{Lux}$ | 0.023 $\frac{\mathbf{1}}{\mathbf{min}}$ | | Corresponds to a protein half-life of 30 min (G. Fritz, S. Dintner, N.S. Treichel, J. Radeck, U. Gerland, T. Mascher, and S. Gebhard, *mBio* 6:e00975, 2015, <https://doi.org/10.1128/mBio.00975-15>) |
| Translation rate | $\beta$ | 10 $\frac{\mathbf{proteins}}{\mathbf{mRNA * min}}$ | | (G. Fritz, S. Dintner, N.S. Treichel, J. Radeck, U. Gerland, T. Mascher, and S. Gebhard, *mBio* 6:e00975, 2015, <https://doi.org/10.1128/mBio.00975-15>) |
| Scaling factor between protein level and luminescence | $\delta$ | 4.56 (+3.1/-1.6)^a^ | | Estimated parameter |
| BcrC module | | | | |
| Relative contribution of BcrC to overall phosphatase activity | $x^{BcrC}$ | 0.63 (+/- 0.095)^b^ | | Estimated parameter |
| Scaling factor for lipid carrier concentrations in *ΔbcrC* | $s^{UPP}$ | 6.2 (+/- 0.7)^b^ | | Estimated parameter |
| Modified model | | | | |
| Michaelis-Menten constant for futile binding of UPP to BceAB | ${K_{M}}^{UPP}$ | 3.38 x 10^4^ $\mu M$ | | Arbitrary choice, expecting an affinity of UPP to BceAB two orders of magnitude lower than the for the native substrate (UPP-BAC) |
